# Age at Menarche and Time Spent in Education: A Mendelian Randomization Study

**DOI:** 10.1007/s10519-017-9862-2

**Published:** 2017-08-09

**Authors:** D. Gill, F. Del Greco M, T. M. Rawson, P. Sivakumaran, A. Brown, N. A. Sheehan, C. Minelli

**Affiliations:** 1 Imperial College London, London, UK; 2 Imperial College Healthcare NHS Trust, London, UK; 3 Institute for Biomedicine, Eurac Research, Bolzano, Italy; 4 University of Leicester, Leicester, UK

**Keywords:** Menarche, Puberty, Education, Educational attainment, Mendelian randomization

## Abstract

Menarche signifies the primary event in female puberty and is associated with changes in self-identity. It is not clear whether earlier puberty causes girls to spend less time in education. Observational studies on this topic are likely to be affected by confounding environmental factors. The Mendelian randomization (MR) approach addresses these issues by using genetic variants (such as single nucleotide polymorphisms, SNPs) as proxies for the risk factor of interest. We use this technique to explore whether there is a causal effect of age at menarche on time spent in education. Instruments and SNP-age at menarche estimates are identified from a Genome Wide Association Study (GWAS) meta-analysis of 182,416 women of European descent. The effects of instruments on time spent in education are estimated using a GWAS meta-analysis of 118,443 women performed by the Social Science Genetic Association Consortium (SSGAC). In our main analysis, we demonstrate a small but statistically significant causal effect of age at menarche on time spent in education: a 1 year increase in age at menarche is associated with 0.14 years (53 days) increase in time spent in education (95% CI 0.10–0.21 years, p = 3.5 × 10^−8^). The causal effect is confirmed in sensitivity analyses. In identifying this positive causal effect of age at menarche on time spent in education, we offer further insight into the social effects of puberty in girls.

**Electronic supplementary material:**

The online version of this article (doi:10.1007/s10519-017-9862-2) contains supplementary material, which is available to authorized users.

## Introduction

Puberty is a time of physiological change in the human body, and its effects extend into the social domains of life (Stattin and Magnusson 1990). In girls, menarche signifies the primary event in puberty. The initiation of the menstrual cycle is associated with reorganization of the self-image, changes in peer relationships, and increased engagement in risk behaviours (Crosnoe 2000). In adolescent females, elevated levels of gonadal hormones follow menarche and influence behavioural development (Schulz and Sisk 2016). Heightened neural plasticity during puberty may predispose to a greater sensitivity to hormones (Piekarski et al. 2016). The significance of menarche as a life-course transition varies with its timing (Schulz and Sisk 2016), and previous observational work has suggested that girls with early puberty have a more difficult journey through school (Cavanagh et al. 2007). While the latter goes on to propose that earlier puberty might be causally associated with less time spent in education, this has not yet been demonstrated. Furthermore, the influence of potentially confounding variables must be reliably excluded to ensure that spurious associations are not interpreted as causal. Previous work has demonstrated age of menarche to be influenced by obesity (Dvornyk and Waqar ul 2012), family size and socio-demographic factors (Chavarro et al. 2004), all of which are also associated with less time spent in education (Winding et al. 2013). Therefore, observational studies on this topic may be limited by confounding, making it difficult to decipher causal effects.

In such situations, the Mendelian randomization (MR) technique can often be used to overcome these limitations by using single nucleotide genetic polymorphisms (SNPs) as instrumental variables (IVs) to explore the direction and magnitude of any causal effect of age at menarche on time spent in education (Davey Smith and Ebrahim 2005). Genes are allocated randomly at the time of conception and are therefore independent of classical confounding. The demonstration that SNPs known to modify age at menarche also modify time spent in education can provide indirect evidence of a causal effect of age at menarche on time spent in education, provided that the necessary assumptions are satisfied (Sheehan et al. 2008). Indeed, such an approach has been previously used to show that earlier menarche causes a higher level of depressive symptoms at 14 years (Sequeira et al. 2016).

Here we use MR to investigate the causal effect of age at menarche on time spent in education. By gaining insight into the effect of age at menarche on time spent in education, we hope to further our understanding of the social implications of this physiological and psychological transition.

## Methods

### SNP-age at menarche association estimates

SNPs for use as instruments in the MR analysis were identified from a GWAS meta-analysis of 57 studies in 182,416 women of European descent, where age at menarche was established by self-reporting, and analyses within each study were adjusted for birth year, to account for secular trends, and genomic control, to account for population stratification (Perry et al. 2014). This identified 122 independent SNPs at 106 genomic loci to be associated with age at menarche (p value < 5 × 10^−8^). We measure the strength of the instruments using the F statistic, which is a function of the magnitude and precision of their genetic effects (Li and Martin 2002; Palmer et al. 2012).

### SNP-time spent in education association estimates

The effects of the 122 instruments on time spent in education were estimated using a GWAS meta-analysis of 118,443 women across 62 studies performed by the Social Science Genetic Association Consortium (SSGAC), the summary estimates for which can be downloaded from http://www.thessgac.org/data (Okbay et al. 2016). The analysis was performed on women, aged 30 years or above, of European descent whose mother tongue was the same as the main language of the country in which they were educated (Okbay et al. 2016). Although study populations were heterogeneous in terms of their educational systems, with different survey questions and data registers used to evaluate time spent in education across studies, comparability was maximized by mapping each major educational qualification on to one of seven categories of the 1997 International Standard Classification of Education (ISCED) of the United Nations Educational, Scientific and Cultural Organization, and then imputing a time spent in education equivalent for each ISCED category (Okbay et al. 2016).

### Mendelian randomization estimates

Individual MR estimates for each of the 122 SNPs were derived using the Wald estimator, which is the ratio of the estimates of the two genetic associations (i.e. SNP-time spent in education estimate over SNP-age at menarche estimate) (Didelez et al. 2010), with standard error derived using the Delta method (Thompson et al. 2016). MR estimates across the individual SNPs were pooled using a fixed-effect inverse-variance weighted (IVW) meta-analysis. This approach assumes an additive model with no interactions for the SNP-age at menarche and SNP-time spent in education relationships.

### Sensitivity analyses

A critical assumption in MR is the absence of pleiotropy—that genetic instruments only modify time spent in education through age at menarche and not by any other independent pathways. In the absence of this condition, MR could produce biased estimates (Sheehan et al. 2008). In the meta-analysis of the 122 MR estimates, the I^2^ index (which we call I^2^_MR_) describes the percentage of total variation in MR estimates across instruments that arises because of heterogeneity rather than chance, and can be used as a proxy for pleiotropy (Del Greco M et al. 2015). We define heterogeneity to be present if I^2^_MR_ > 25%. To address pleiotropy and other possible sources of bias in this work, further sensitivity analyses were performed:

1. *MR-Egger* This is an adaptation of Egger regression applied to the context of two-sample MR that uses multiple genetic variants (Bowden et al. 2015). The MR-Egger approach can be used to provide unbiased results in the presence of pleiotropic instruments under the assumption that the magnitude of pleiotropic effects is independent of the magnitude of the corresponding SNP-age at menarche effects (Bowden et al. 2015). The degree of heterogeneity in the SNP-age at menarche estimates generated by the different instruments, as measured using the I^2^ statistic (called I^2^_GX_ here), is used to quantify any potential bias arising in the MR-Egger analysis due to measurement error. An I^2^_GX_ estimate close to 100% would suggest that such a phenomenon is not creating bias, as greater heterogeneity reduces regression dilution with MR-Egger (Bowden et al. 2016b).
2. *Weighted median estimator* Used as a further sensitivity analysis here, this approach orders the MR estimates generated using each instrument separately by the inverse of their variances; selecting the median result provides a single MR estimate, with confidence intervals generated using a parametric bootstrap method (Bowden et al. 2016a). The weighted median estimator does not require that the magnitude of any pleiotropic effects of the instruments are uncorrelated to their effects on the intermediate phenotype, as MR-Egger does, but instead assumes that at least half of the instruments are valid (Bowden et al. 2016b).
3. *Exclusion of instruments also associated with body mass index (BMI)* Age at menarche has been previously demonstrated to be associated with obesity (Dvornyk and Waqar ul 2012). If BMI is also associated with time spent in education, then any instruments for age at menarche that are also associated with BMI might be introducing pleiotropic effects by this mechanism. Sensivity analysis was therefore also performed by repeating the MR-analysis using the fixed-effect IVW meta-analysis approach with the exclusion of SNPs also associated with BMI at genome-wide significance level (Supplementary Table 1).
4. *Unweighted allele score* The use of SNP-age at menarche estimates generated from the GWAS discovery analysis rather than the replication analysis, which had a sample size 20 times smaller (8689 vs. 182,416) (Perry et al. 2014), may result in the possible upward bias that is typical of discovery stage results (Ioannidis et al. 2001). MR analysis using a fixed-effect IVW meta-analysis of SNP-time spent in education association estimates across the 122 SNPs, which is equivalent to an “unweighted allele score” (Charoen et al. 2016), is not affected by this form of bias and is therefore used here as a sensitivity analysis.
5. *SNP-time spent in education estimates for men using an unweighted allele score* As a check that the SNP-time spent in education association observed in women is indeed driven by mediation through age at menarche and not via alternative pathways, as implied by our instrumental variable assumptions, we estimate this association in men using an unweighted allele score for the 122 age at menarche instruments. Since men do not undergo menarche, the exposure under investigation, lack of any association in men would provide further evidence that an association in women is due to a causal effect of age at menarche on time spent in education. These SNP-time spent in education estimates were obtained from a GWAS meta-analysis of 147,474 men also performed by the SSGAC, the summary estimates for which can be downloaded from http://www.thessgac.org/data (Okbay et al. 2016).

All analyses were performed using Stata 14 (StataCorp LP) and R version 3.3.2 (R Core Team).

## Results

Supplementary Tables 2 and 3 report individual SNP estimates of the per-allele effects on age at menarche (years) and time spent in education for women (standard deviation change in time spent in education, measured in years), respectively, while Supplementary Table 4 reports individual SNP MR estimates for the causal effect of age at menarche on time spent in education (standard deviation change in time spent in education, measured in years, per year increase in age at menarche). The considered SNPs are all strong instruments for age at menarche, with F statistics ranging from 25 to 576 (Supplementary Table 2), which are all greater than the recommended threshold of 10 (Lawlor et al. 2008).

The fixed-effect IVW meta-analysis of all 122 SNPs shows a statistically significant causal effect of age at menarche on time spent in education: a 1 year increase in age at menarche is associated with a 0.04 standard deviation units increase in time spent in education (95% CI 0.03–0.06), with a p value of 3.5 × 10^−8^ (Supplementary Fig. 1). With the standard deviation of time in education reported as 3.6 years (Okbay et al. 2016), this equates to 0.14 years (53 days, 95% CI 0.10–0.21 years). However, there is evidence of pleiotropy among instruments, with a between-instrument I^2^_MR_ of 48% (95% CI 36–58%). Further sensitivity analyses were performed, and MR-Egger regression analysis estimated that a 1 year increase in age at menarche is associated with 0.10 standard deviation increase in time spent in education (95% CI 0.03–0.18, p = 0.01) (Supplementary Fig. 1). The I^2^_GX_ statistic is 85%, suggesting that there is no major evidence of measurement error biasing MR-Egger analysis (Bowden et al. 2016b). The MR-Egger intercept is −0.002 (95% CI −0.006 to 0.001, p = 0.139), suggesting no evidence of directional pleiotropy (Bowden et al. 2015). Supplementary Fig. 2 shows the funnel plot of the minor allele frequency corrected GX estimates by the GY/GX estimates; there is no major asymmetry around the fixed-effect IVW meta-analysis causal estimate (dashed red line) to suggest directional pleiotropy.

The weighted median approach estimates that a 1 year increase in age at menarche is associated with 0.05 standard deviation increase in time spent in education (95% CI 0.03–0.07, p = 7.34 × 10^−5^) (Supplementary Fig. 1). Fixed-effect IVW meta-analysis after excluding the 12 SNPs associated with BMI (Supplementary Table 1) also shows a statistically significant causal effect of age at menarche on time spent in education: a 1 year increase in age at menarche is associated with 0.05 standard deviation increase in time spent in education (95% CI 0.03–0.06, p = 1.74 × 10^−8^), although evidence of pleiotropy persists (I^2^_MR_ 47%, 95% CI 34–58%). Thus, removing these 12 SNPs that are associated with BMI did not have any major effect on the results obtained, and for this reason, the results of the original IVW approach are reported as the main analysis.

Use of an unweighted allele score for the 122 instruments in women shows a statistically significant positive association with time spent in education (p = 3.61 × 10^−7^). This is reassuring that the causal effect of age at menarche on time spent in education shown by our main analysis is not attributable to bias due to use of SNP-age at menarche estimates from discovery stage results. The unweighted allele score is only used here to test for a causal effect of age at menarche on time spent in education and not to estimate the magnitude of this effect.

Supplementary Table 5 reports individual SNP estimates for the per-allele effect of the 122 age at menarche instruments on time spent in education for men. The unweighted allele score for the 122 age at menarche instruments using SNP-time spent in education estimates for men is not significant (p = 0.72), thus strengthening our belief that the observed association in women is driven by mediation through age at menarche.

In summary, all sensitivity analyses support our findings of a statistically significant causal effect of age at menarche on time spent in education.

## Discussion

We have used MR to investigate the causal effect of age at menarche on time spent in education. Under the required assumptions, this technique circumvents the classical confounding seen in observational studies, and our main (fixed-effect IVW meta-analysis of all 122 SNPs) analysis suggests that for every year increase in age at menarche, women spend an extra 53 days in education on average (95% CI 37–78 days). The main limitation of the MR approach is possible bias due to pleiotropic instruments, and we have addressed this using several sensitivity analyses.

The physical, behavioural and cognitive aspects of development that are associated with puberty vary in their timing. A lower age at menarche has been hypothesized to result in earlier physical and physiological development, but without matching levels of cognitive and behavioural development. This delay can lead to inadequate coping strategies, greater risk-taking behaviour, lower social competence, and higher rates of internalizing and affective disorders (Brooks-Gunn and Warren 1989; Stice et al. 2004; Westling et al. 2012), all of which may culminate in less time spent in education.

Previous work has highlighted the adverse effects of early puberty on self-perception, peer-relationships and risk-taking behaviours in girls, with consequent effects on performance in school (Correll 2001; Crosnoe 2000; Graber 2013; Mendle et al. 2007). However, there has been comparatively little work directly exploring the effect of age at menarche on time spent in education. One survey-based observational study performed by Koivusilta et al. investigating how age at menarche predicts time spent in education separately considered samples of 903, 1430 and 1584 Finnish girls aged 12 years, 14 years and 16 years respectively (Koivusilta and Rimpela 2004). This work measured the timing of menarche as early (age 11 years or younger), average (age 12 or 13 years) or late (age 14 years or older), with time spent in education divided into categories of 9–10, 11–12, 13–15 and 16–18 years. While ordinal logistic regression did not identify any effect of age at menarche on time spent in education, it is possible that use of ordered categorical variables, rather than continuous ones, might have resulted in loss of power to detect the small effect size identified in our analysis. Furthermore, such observational work is also susceptible to the effects of confounding, such as from socio-demographic factors (Koivusilta and Rimpela 2004), the direction of which is hard to predict (away from the null or towards the null).

There are a number of possible sources of bias in our work. The age at menarche estimates used were self-reported and are therefore susceptible to recall bias (Perry et al. 2014). Furthermore, the different studies used to generate SNP-time spent in education association results were spread out over decades, with birth years ranging from 1901 to 1989 (Okbay et al. 2016). The social environment is likely to have changed over this period, making the MR analysis susceptible to varying SNP-environment interactions (Brennan 2004). Finally, although the use of SNP-age at menarche estimates generated from the GWAS discovery analysis may result in upward bias (“winner’s curse”), we have shown that a causal effect remains when testing it using an unweighted allele score, which is not affected by this “winner’s curse”.

In summary, we have used the MR approach to tackle traditional confounding in investigating the effect of age at menarche on time spent in education. We demonstrate a small positive causal effect, which offers further insight into the effects of puberty in girls. Given the significance of education on future life course, to include effects on health, this finding provides further insight into the social, psychological and physiological factors that determine time in education (Brennan 2004; Kingston et al. 2003; Li and Powdthavee 2015).

## Electronic supplementary material

Below is the link to the electronic supplementary material.

**Supplementary Figure 1**. Scatter plot of the SNP-time spent in education (GY; standard deviation change in time, years, spent in education; y-axis) and SNP-age at menarche (GX; years; x-axis) estimates for all 122 SNPs. The red line depicts the IVW meta-analysis estimate, the dashed blue line the MR-Egger estimate and the dashed black line the weighted median estimator. (BMP 1122 KB)

**Supplementary Figure 2**. Funnel plot of 1/standard error of MR estimate (y-axis) by the MR estimate (x-axis), to highlight any evidence of directional pleiotropy (Bowden et al., 2015; Bowden et al., 2017). There is no major asymmetry around the fixed-effect IVW meta-analysis causal estimate (dashed red line) to suggest directional pleiotropy. The blue line depicts the MR-Egger causal estimate. (BMP 1181 KB)

Supplementary material 3 (DOCX 648 KB)

Supplementary material 4 (XLSX 20 KB)

Supplementary material 5 (XLSX 13 KB)

Supplementary material 6 (XLSX 14 KB)

Supplementary material 7 (XLSX 15 KB)
