# Supplementary figures and images for: Age at Menarche and Time Spent in Education: A Mendelian Randomization Study
Source: Behav Genet. 2017 Aug 9;47(5):480–5. doi: 10.1007/s10519-017-9862-2 (PMC5574970; doi:10.1007/s10519-017-9862-2)

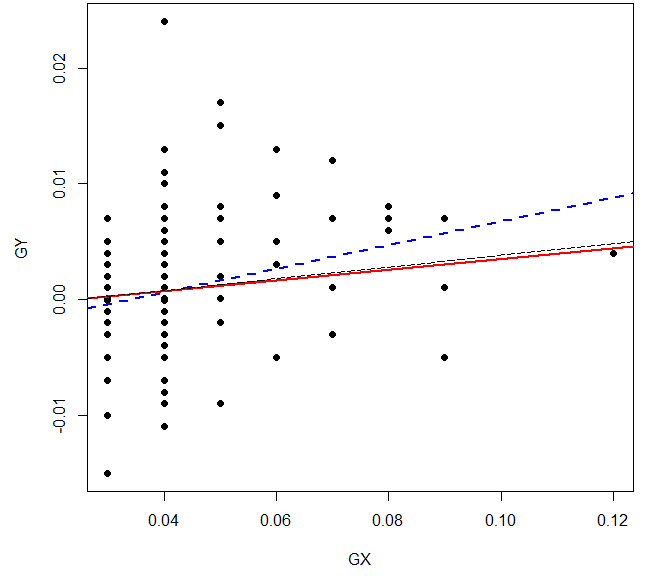

Supplement: Supplementary file 1 — Supplementary Figure 1. Scatter plot of the SNP-time spent in education (GY; standard deviation change in time, years, spent in education; y-axis) and SNP-age at menarche (GX; years; x-axis) estimates for all 122 SNPs. The red line depicts the IVW meta-analysis estimate, the dashed blue line the MR-Egger estimate and the dashed black line the weighted median estimator. (BMP 1122 KB) [file 10519_2017_9862_MOESM1_ESM.bmp]

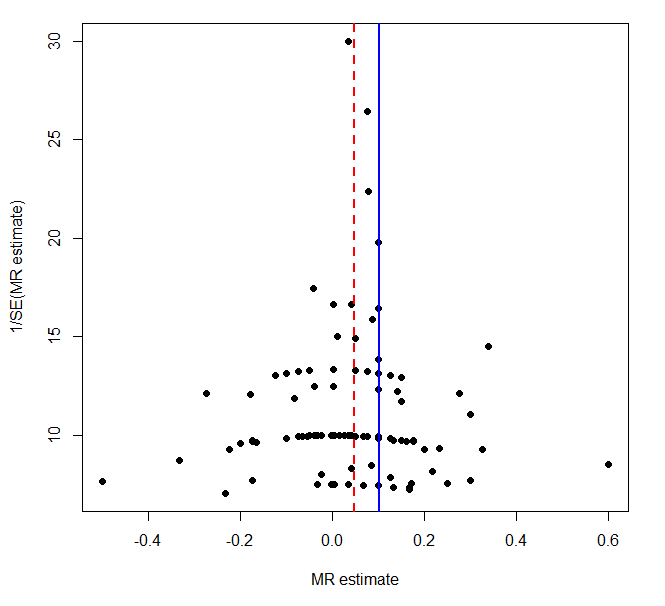

Supplement: Supplementary file 2 — Supplementary Figure 2. Funnel plot of 1/standard error of MR estimate (y-axis) by the MR estimate (x-axis), to highlight any evidence of directional pleiotropy (Bowden et al., 2015; Bowden et al., 2017). There is no major asymmetry around the fixed-effect IVW meta-analysis causal estimate (dashed red line) to suggest directional pleiotropy. The blue line depicts the MR-Egger causal estimate. (BMP 1181 KB) [file 10519_2017_9862_MOESM2_ESM.bmp]
