# Supplementary material for: Age at Menarche and Time Spent in Education: A Mendelian Randomization Study
Source: Behav Genet. 2017 Aug 9;47(5):480–5. doi: 10.1007/s10519-017-9862-2 (PMC5574970; doi:10.1007/s10519-017-9862-2)
Supplement: Supplementary file 3 — Supplementary material 3 (DOCX 648 KB) [file 10519_2017_9862_MOESM3_ESM.docx]

**Supplementary Table 1.** Evidence of association of the 122 SNPs (and SNPs highly correlated with them, linkage disequilibrium r^2^>0.8) with body mass index (BMI) (information retrieved from Phenoscanner, available at: <http://www.phenoscanner.medschl.cam.ac.uk/phenoscanner>) ([Staley et al., 2016](#_ENREF_10)).

| **SNP** | **Gene** | **Studies identifying an association with BMI** |
| --- | --- | --- |
| rs10144321 | WDR25 | / |
| rs1038903 |  | / |
| rs10423674 | CRTC1 | / |
| rs10453225 |  | / |
| rs10739221 |  | / |
| rs10789181 | DNAJC6 | / |
| rs1079866 |  | / |
| rs10816359 |  | / |
| rs10895140 | TRPC6 | / |
| rs10938397 |  | ([Berndt et al., 2013](#_ENREF_1); [Gaulton et al., 2015](#_ENREF_2); [Graff et al., 2013](#_ENREF_3); [Randall et al., 2013](#_ENREF_8); [Speliotes et al., 2010](#_ENREF_9); [Willer et al., 2009](#_ENREF_15); [Winkler et al., 2015](#_ENREF_16)) |
| rs10980854 |  | / |
| rs10980921 |  | / |
| rs11022756 | ARNTL | / |
| rs11165924 | DPYD | / |
| rs11215400 | CADM1 | / |
| rs1129700 | ASPHD1,  KCTD13 | / |
| rs11578152 |  | / |
| rs11715566 |  | / |
| rs11767400 | CADPS2 | / |
| rs11792861 | MIR32,  TMEM245 | / |
| rs12148769 |  | / |
| rs12446632 |  | ([Berndt et al., 2013](#_ENREF_1); [Gaulton et al., 2015](#_ENREF_2); [Randall et al., 2013](#_ENREF_8); [Speliotes et al., 2010](#_ENREF_9); [Winkler et al., 2015](#_ENREF_16)) |
| rs12472911 | LRP1B | / |
| rs1254337 | C14orf39 | / |
| rs12571664 |  | / |
| rs12607903 | DLGAP1 | / |
| rs12915845 |  | / |
| rs13053505 |  | / |
| rs13067731 |  | / |
| rs13135934 | SMARCAD1 | / |
| rs13179411 | JADE2 | / |
| rs13196561 |  | / |
| rs1324913 | KLF12 | / |
| rs1364063 |  | / |
| rs1400974 |  | / |
| rs1461503 |  | / |
| rs1469039 | KCNK9 | / |
| rs1532331 |  | / |
| rs16860328 | TRA2B | / |
| rs16896742 |  | / |
| rs16918254 |  | / |
| rs16918636 |  | / |
| rs17086188 |  | / |
| rs17171818 | KDM3B | / |
| rs17233066 |  | / |
| rs17236969 |  | / |
| rs17266097 | SATB2 | / |
| rs1874984 | ADARB2 | / |
| rs1915146 | CTBP2 | / |
| rs1958560 | FUT8 | / |
| rs2063730 | GAB2 | / |
| rs2137289 | SKOR2 | / |
| rs2153127 |  | / |
| rs2274465 | KDM4A | / |
| rs239198 | ASCC3 | / |
| rs244293 | STXBP4 | / |
| rs246185 | MIR193BHG | / |
| rs2479724 | BYSL | / |
| rs251130 | STARD4-AS1 | / |
| rs2600959 |  | / |
| rs268067 |  | / |
| rs2687729 | EEFSEC | / |
| rs2688325 | CSMD1 | / |
| rs2836950 | BRWD1 | / |
| rs2947411 |  | ([Berndt et al., 2013](#_ENREF_1); [Gaulton et al., 2015](#_ENREF_2); [Randall et al., 2013](#_ENREF_8); [Speliotes et al., 2010](#_ENREF_9); [Winkler et al., 2015](#_ENREF_16)) |
| rs3101336 |  | ([Berndt et al., 2013](#_ENREF_1); [Gaulton et al., 2015](#_ENREF_2); [Randall et al., 2013](#_ENREF_8); [Speliotes et al., 2010](#_ENREF_9); [Thorleifsson et al., 2009](#_ENREF_11); [Wheeler et al., 2013](#_ENREF_14); [Winkler et al., 2015](#_ENREF_16)) |
| rs3733631 | TACR3 | / |
| rs3743266 | RORA-AS1,  RORA | / |
| rs4369815 |  | / |
| rs466639 | RXRG | / |
| rs4756059 |  | / |
| rs4840086 |  | / |
| rs4875053 |  | / |
| rs4895808 | CENPW | / |
| rs4929947 | TRIM66 | ([Gaulton et al., 2015](#_ENREF_2); [Winkler et al., 2015](#_ENREF_16)) |
| rs543874 |  | ([Berndt et al., 2013](#_ENREF_1); [Gaulton et al., 2015](#_ENREF_2); [Graff et al., 2013](#_ENREF_3); [Guo et al., 2013](#_ENREF_4); [Monda et al., 2013](#_ENREF_6); [Randall et al., 2013](#_ENREF_8); [Speliotes et al., 2010](#_ENREF_9); [Winkler et al., 2015](#_ENREF_16)) |
| rs6009583 |  | / |
| rs6427782 |  | / |
| rs652260 | EVI5L | / |
| rs6555855 |  | / |
| rs6563739 | COG6,  MIR4305 | / |
| rs6747380 | CCDC85A | / |
| rs6758290 |  | / |
| rs6762477 | RBM6 | / |
| rs6770162 |  | / |
| rs6933660 |  | / |
| rs6938574 | PTPRK | / |
| rs6964833 | GTF2I | / |
| rs7037266 | KDM4C | / |
| rs7103411 | BDNF-AS,  BDNF | ([Gaulton et al., 2015](#_ENREF_2); [Randall et al., 2013](#_ENREF_8); [Speliotes et al., 2010](#_ENREF_9); [Winkler et al., 2015](#_ENREF_16)) |
| rs7104764 | SIRT3 | / |
| rs7138803 |  | ([Berndt et al., 2013](#_ENREF_1); [Gaulton et al., 2015](#_ENREF_2); [Guo et al., 2013](#_ENREF_4); [Randall et al., 2013](#_ENREF_8); [Speliotes et al., 2010](#_ENREF_9); [Thorleifsson et al., 2009](#_ENREF_11); [Winkler et al., 2015](#_ENREF_16)) |
| rs7141210 |  | / |
| rs7215990 |  | / |
| rs7463166 | CSMD1 | / |
| rs7514705 | FPGT-TNNI3K,  TNNI3K | ([Gaulton et al., 2015](#_ENREF_2); [Randall et al., 2013](#_ENREF_8); [Speliotes et al., 2010](#_ENREF_9); [Winkler et al., 2015](#_ENREF_16)) |
| rs7642134 |  | / |
| rs7647973 | DAG1 | / |
| rs7701886 |  | ([Monda et al., 2013](#_ENREF_6)) |
| rs7759938 |  | / |
| rs7821178 |  | / |
| rs7828501 | CSMD1 | / |
| rs7853970 |  | / |
| rs7865468 | PTPRD | / |
| rs7955374 |  | / |
| rs8032675 | MAP2K5 | ([Gaulton et al., 2015](#_ENREF_2); [Randall et al., 2013](#_ENREF_8)) |
| rs8050136 | FTO | ([Berndt et al., 2013](#_ENREF_1); [Gaulton et al., 2015](#_ENREF_2); [Heard-Costa et al., 2009](#_ENREF_5); [Paternoster et al., 2011](#_ENREF_7); [Randall et al., 2013](#_ENREF_8); [Speliotes et al., 2010](#_ENREF_9); [Thorleifsson et al., 2009](#_ENREF_11); [Timpson et al., 2009](#_ENREF_12); [Wan et al., 2011](#_ENREF_13); [Winkler et al., 2015](#_ENREF_16)) |
| rs852069 |  | / |
| rs889122 | OLFM2 | / |
| rs900400 |  | / |
| rs913588 | KDM4C | / |
| rs929843 | PDXDC2P | / |
| rs9321659 |  | / |
| rs939317 | EIF4G1 | / |
| rs9447700 |  | / |
| rs9475752 | DST | / |
| rs951366 | NUCKS1 | / |
| rs9560113 |  | / |
| rs9635759 |  | / |
| rs9647570 | TENM2 | / |
| rs9849248 |  | / |
| rs988913 | FAM83B | / |

**References**

Berndt, S. I., Gustafsson, S., Magi, R., Ganna, A., Wheeler, E., Feitosa, M. F., . . . Ingelsson, E. (2013). Genome-wide meta-analysis identifies 11 new loci for anthropometric traits and provides insights into genetic architecture. *Nat Genet, 45*(5), 501-512. doi: 10.1038/ng.2606

Gaulton, K. J., Ferreira, T., Lee, Y., Raimondo, A., Magi, R., Reschen, M. E., . . . Meta-analysis, C. (2015). Genetic fine mapping and genomic annotation defines causal mechanisms at type 2 diabetes susceptibility loci. *Nat Genet, 47*(12), 1415-1425. doi: 10.1038/ng.3437

Graff, M., Ngwa, J. S., Workalemahu, T., Homuth, G., Schipf, S., Teumer, A., . . . Berndt, S. I. (2013). Genome-wide analysis of BMI in adolescents and young adults reveals additional insight into the effects of genetic loci over the life course. *Hum Mol Genet, 22*(17), 3597-3607. doi: 10.1093/hmg/ddt205

Guo, Y., Lanktree, M. B., Taylor, K. C., Hakonarson, H., Lange, L. A., Keating, B. J., & Consortium, I. K. S. a. B. (2013). Gene-centric meta-analyses of 108 912 individuals confirm known body mass index loci and reveal three novel signals. *Hum Mol Genet, 22*(1), 184-201. doi: 10.1093/hmg/dds396

Heard-Costa, N. L., Zillikens, M. C., Monda, K. L., Johansson, A., Harris, T. B., Fu, M., . . . North, K. E. (2009). NRXN3 is a novel locus for waist circumference: a genome-wide association study from the CHARGE Consortium. *PLoS Genet, 5*(6), e1000539. doi: 10.1371/journal.pgen.1000539

Monda, K. L., Chen, G. K., Taylor, K. C., Palmer, C., Edwards, T. L., Lange, L. A., . . . Haiman, C. A. (2013). A meta-analysis identifies new loci associated with body mass index in individuals of African ancestry. *Nat Genet, 45*(6), 690-696. doi: 10.1038/ng.2608

Paternoster, L., Evans, D. M., Nohr, E. A., Holst, C., Gaborieau, V., Brennan, P., . . . Sorensen, T. I. (2011). Genome-wide population-based association study of extremely overweight young adults--the GOYA study. *PLoS One, 6*(9), e24303. doi: 10.1371/journal.pone.0024303

Randall, J. C., Winkler, T. W., Kutalik, Z., Berndt, S. I., Jackson, A. U., Monda, K. L., . . . Heid, I. M. (2013). Sex-stratified genome-wide association studies including 270,000 individuals show sexual dimorphism in genetic loci for anthropometric traits. *PLoS Genet, 9*(6), e1003500. doi: 10.1371/journal.pgen.1003500

Speliotes, E. K., Willer, C. J., Berndt, S. I., Monda, K. L., Thorleifsson, G., Jackson, A. U., . . . Loos, R. J. (2010). Association analyses of 249,796 individuals reveal 18 new loci associated with body mass index. *Nat Genet, 42*(11), 937-948. doi: 10.1038/ng.686

Staley, J. R., Blackshaw, J., Kamat, M. A., Ellis, S., Surendran, P., Sun, B. B., . . . Butterworth, A. S. (2016). PhenoScanner: a database of human genotype-phenotype associations. *Bioinformatics, 32*(20), 3207-3209. doi: 10.1093/bioinformatics/btw373

Thorleifsson, G., Walters, G. B., Gudbjartsson, D. F., Steinthorsdottir, V., Sulem, P., Helgadottir, A., . . . Stefansson, K. (2009). Genome-wide association yields new sequence variants at seven loci that associate with measures of obesity. *Nat Genet, 41*(1), 18-24. doi: 10.1038/ng.274

Timpson, N. J., Lindgren, C. M., Weedon, M. N., Randall, J., Ouwehand, W. H., Strachan, D. P., . . . McCarthy, M. I. (2009). Adiposity-related heterogeneity in patterns of type 2 diabetes susceptibility observed in genome-wide association data. *Diabetes, 58*(2), 505-510. doi: 10.2337/db08-0906

Wan, E. S., Cho, M. H., Boutaoui, N., Klanderman, B. J., Sylvia, J. S., Ziniti, J. P., . . . investigators, C. G. (2011). Genome-wide association analysis of body mass in chronic obstructive pulmonary disease. *Am J Respir Cell Mol Biol, 45*(2), 304-310. doi: 10.1165/rcmb.2010-0294OC

Wheeler, E., Huang, N., Bochukova, E. G., Keogh, J. M., Lindsay, S., Garg, S., . . . Farooqi, I. S. (2013). Genome-wide SNP and CNV analysis identifies common and low-frequency variants associated with severe early-onset obesity. *Nat Genet, 45*(5), 513-517. doi: 10.1038/ng.2607

Willer, C. J., Speliotes, E. K., Loos, R. J., Li, S., Lindgren, C. M., Heid, I. M., . . . Genetic Investigation of, A. T. C. (2009). Six new loci associated with body mass index highlight a neuronal influence on body weight regulation. *Nat Genet, 41*(1), 25-34. doi: 10.1038/ng.287

Winkler, T. W., Justice, A. E., Graff, M., Barata, L., Feitosa, M. F., Chu, S., . . . Loos, R. J. (2015). The Influence of Age and Sex on Genetic Associations with Adult Body Size and Shape: A Large-Scale Genome-Wide Interaction Study. *PLoS Genet, 11*(10), e1005378. doi: 10.1371/journal.pgen.1005378
